# Reconciling Hard Skills and Soft Skills in a Common Framework: The Generic Skills Component Approach

**DOI:** 10.3390/jintelligence11060107

**Published:** 2023-06-01

**Authors:** Jeremy Lamri, Todd Lubart

**Affiliations:** LaPEA, Université Paris Cité & Univ Gustave Eiffel, F-92100 Boulogne-Billancourt, France

**Keywords:** skills, soft skills, hard skills, cognition, conation, affection

## Abstract

The distinction between hard and soft skills has long been a topic of debate in the field of psychology, with hard skills referring to technical or practical abilities, and soft skills relating to interpersonal capabilities. This paper explores the generic composition of any skill, proposing a unified framework that consists of five distinct components: knowledge, active cognition, conation, affection, and sensory-motor abilities. Building upon previous research and theories, such as Hilgard’s “Trilogy of Mind”, the generic skill components approach aims to provide a comprehensive understanding of the structure and composition of any skill, whether hard or soft. By examining these components and their interactions, we can gain a more in-depth understanding of the nature of skills and their development. This approach has several potential applications and implications for various fields, including education, training, and workplace productivity. Further research is needed to refine and expand upon the generic skill components theory, exploring the interactions between the different components, as well as the impact of contextual factors on skill development and use.

## 1. Introduction

In today’s complex, interconnected world, the importance of having a diverse set of skills for success is undeniable. The ability to define, develop and utilise one’s skills is considered a vital part of personal and professional success. This success depends heavily on the acquisition and maintenance of both soft and hard skills. In the modern workforce, employers are searching for the perfect candidate, the one who can bring a combination of skills to the table. Indeed, skills can generally be divided into two main categories—hard skills and soft skills. Hard skills refer to technical or practical abilities, such as programming languages, engineering, accounting, and other occupational skills, whereas soft skills are interpersonal capabilities, such as communication, problem-solving, and emotional intelligence (Cimatti 2016; Laker and Powell 2011).

Although these two types of skills are often categorised separately, it is important to understand their interdependence, as well as their contributions to certain areas of expertise. In recent years, there has been increasing recognition of the importance of soft skills in many areas, including education and business (Andrews and Higson 2008; Succi and Canovi 2020). The so-called “soft skill revolution” has seen a growing interest in developing and assessing these skills, as organisations have become increasingly aware of their value in the workplace. Yet, there is still some debate about what constitutes a soft skill, and to what extent hard skills remain essential for success. Despite the acknowledged value of soft skills, the lack of a standard definition or systematic approach to measuring and assessing these skills poses a challenge when attempting to review and compare them (Dede 2010; Robles 2012; Rasipuram and Jayagopi 2020).

Even before challenging the concept of soft skills, there is the question of what a “skill” is, and how to develop certain skills, as it remains an ongoing area of research for psychologists and educators. Whereas the study of skills has traditionally been associated with individual traits such as intelligence and talent, an emerging field of inquiry suggests that the composition of any skill is made up of several core elements. Overall, skills are an important foundation for development, yet much research is needed to understand better the generic components of skills. Although soft skills and hard skills seem very different in the way they are used and observed, what actually makes them inherently different? If both are actually skills, they may have more in common than it seems. In recent years, research into the generic composition of any skill, and the relationship between soft skills and hard skills, has gained increased interest due to its implications for workplace productivity.

Researchers have identified that any workplace skill requires a combination of hard and soft skills (van der Vleuten et al. 2019; Lyu and Liu 2021). They have also elucidated that there are shared components between hard and soft skills which could be seen as the bridge between them (Pieterse and Van Eekelen 2016; Kuzminov et al. 2019). This presents an interesting opportunity for educators and trainers to develop individuals in an integrated manner, allowing for an understanding of both technical and non-technical components of skills.

This paper explores the generic composition of any skill and the common ground between soft skills and hard skills. Hilgard’s “Trilogy of Mind” (1980) provides useful insights into the debate, by suggesting that all skills—whether hard or soft—can be understood in terms of three distinct components: cognition, conation, and affection. In this article, we will discuss how Hilgard’s theory can be applied in order to describe the composition of any skill, and argue that, theoretically, there is no difference between soft and hard skills, opening the way to a generic skills framework.

## 2. Critical Literature Review

### 2.1. Definition of Skill

As the distinction between soft and hard skills is not standardised, it is important to consider different definitions of “skill” for the purposes of this article. Skill is a multifaceted concept that has been studied extensively in the scientific literature (Vallas 1990; Clarke and Winch 2006; Green 2011). According to the definition of the 2023 Merriam-Webster dictionary, a skill is “the ability to use one’s knowledge effectively and readily in execution or performance” (Merriam-Webster n.d.). It describes the ability that develops from practice, training, and experience to perform a specific task to a certain standard. It has been subject to considerable examination by researchers across different disciplines, including psychology, neuroscience, education and sports science (Gagné and Fleishman 1959; Frese and Stewart 1984; Bo et al. 2008).

Boyatzis (1982) defined “skill” as “an underlying characteristic of a person that has a causal relationship with their average or superior performance in a given function”. In more concrete terms, “skill” refers to an individual’s ability to accomplish tasks by utilising appropriate resources, including those acquired through training or previous experience (Le Boterf 2000). A skill can be conceptualised as specific know-how that is pertinent to a given situation, resulting in the combination of knowledge, other mental abilities and physical strength, agility, coordination, and motor abilities (Green 2011). This definition provides a clear understanding of the underlying competencies and knowledge necessary to successfully carry out any task, regardless of whether it concerns soft or hard skills. In addition, the success of skills is partially dependent on the direct content of the tasks, abilities, values, interests, and the environment of the individual (Le Boterf 2000).

As noted by DeKeyser (2020), the term “skill” encompasses the ability to process and understand information, interpret, and use it in order to complete a task. It involves both cognitive and motor abilities, which together form a basis for mastery (Roebers et al. 2014; Van der Fels et al. 2015). Both require knowledge and the ability to store and recall information, as well as the ability to interpret and apply it correctly. Through practice and repetition, skills become increasingly automatic and rapid, and proficiency is observed. 

As such, “skill” can be seen as the ability to retrieve knowledge and apply it to a task in a proficient manner. Cognitive factors include working memory, various forms of reasoning, and problem-solving (Carroll 2003). Motor abilities include factors such as coordination, muscle and joint strength, and speed (Zajac 1993). In more psychological terms, they can be seen as a component of behavioural abilities. When including motor abilities, the dyad created by cognitive and behavioural components plays an important role in the development and refinement of skills. This is an important concept to recognise when considering the notion of skill, as both the ability to understand and interpret knowledge, as well as the application of what has been learnt are essential for skill development. In conclusion, a skill is an ability that is refined with training, technique, and experience. It is noted to involve a combination of cognitive and behavioural components which interact to allow the effective completion of a given task. 

A wide range of skills have been studied, such as motor skills, sensory and perceptual skills, cognitive skills, and social skills (Fischer 1980). Motor skills are defined as the ability to control and coordinate the movements and actions of the body (Newell 1991). Sensory and perceptual skills involve the ability to receive, interpret, and act upon sensory information, such as visual, auditory, and tactile data (Karni and Bertini 1997). Cognitive skills encompass the ability to think logically, problem-solve, and make decisions, whereas social skills involve the ability to interact and communicate effectively with others (Patterson 2008). Overall, skills are multifaceted constructs that enable humans to continue to grow and learn in a variety of contexts, through general practice and experience, as well as through the development of specific tasks and strategies. 

### 2.2. Definitions and Characteristics of Hard Skills

Hard skills refer to technical, tangible, and quantifiable abilities related to the use of equipment for a specific job, such as driving a car, computer programming, or welding (Lyu and Liu 2021). Hard skills are typically acquired through training and education and are a requisite for performing job duties. They are necessary for specific tasks within an industry that requires specific expertise and proficiency, such as welding, accounting, and using a 3-D printer. As researchers note, hard skills are also differently defined along the lines of work and education. A person with a background in computer science may define hard skills as the technical abilities required for software development, whereas someone with a background in design may define hard skills as the artistic abilities needed for graphic design. The importance of hard skills has long been acknowledged in the workplace, especially because the manipulation of these skills often leads to measurable performance outcomes (Rainsbury et al. 2002; Hendarman and Cantner 2018). Consequently, they are usually emphasised during recruitment processes and have been found to play a determining role in the hiring decisions of employers (Bishop 2017; Huber 2018). Actually, both motivation and hard skills play an important role in positive job performance (Hendarman and Cantner 2018).

### 2.3. Definitions and Characteristics of Soft Skills

In 1972, the term “soft skills” was first used by the researcher Paul G. Whitmore, during a training conference in Texas for the US Army Continental Army Command (CONARC). Whitmore used the term “soft skills” to refer to crucial job-related skills that involve little or no interaction with machines (CONARC 1972, cited in Parlamis and Monnot 2019). They may as well be considered behaviours that a person must mobilise in order to reach a given objective competently (Tate 1995). Considering the context of hard skills, soft skills are non-technical abilities that are harder to measure and quantify (Kantrowitz 2005; Byrne et al. 2020). Soft skills involve personal, interpersonal, and intrapersonal abilities that are essential in the workplace (Dell’Aquila et al. 2017). Examples of soft skills include emotional intelligence, communication, creativity, problem-solving, team building, and stress management (Martins et al. 2020).

Unlike hard skills, soft skills tend not to be acquired through formal education and training and often require dedication, self-reflection, and self-improvement (Chell and Athayde 2011; Wisshak and Hochholdinger 2020). This does not mean that hard skills do not require these same qualities, however, the probability of systematic acquisition seems less predictable for soft skills, and more related to personal qualities, as their use will be specific to every person. Furthermore, soft skills are typically more developed through social experience, which is why they are often referred to as “people skills” (Levasseur 2013). 

There are many different terminologies when referring to soft skills, such as social competencies, interpersonal skills, or even emotional intelligence (Matteson et al. 2016). Social competencies encompass a broader range of abilities that enable individuals to navigate effectively interpersonal situations, build and maintain relationships, and work well with others. These competencies include communication, teamwork, adaptability, and cultural awareness (Rychen and Salganik 2003). Interpersonal skills refer to the abilities needed to effectively interact, communicate, and collaborate with others. These skills include active listening, empathy, conflict resolution, and negotiation (Spencer and Spencer 1993). Emotional intelligence encompasses the ability to recognise, understand, and manage one’s own emotions and the emotions of others. It is closely related to interpersonal skills and includes self-awareness, self-regulation, motivation, empathy, and social skills (Goleman 1995; Mayer et al. 2008).

With over 119 labels identified in the literature in 600 publications about soft skills over the past 50 years (Joie-La Marle et al. 2022), numerous frameworks have been created to categorise and understand them. Depending on the approach, these frameworks deal with social skills, emotional skills, cognitive skills, or all of them. Their main interest is generally to delineate critical skills needed for the future of work, which is the reason why the field of education is where most frameworks are created. Researchers, schools, and even international organisations have created their own soft skills frameworks. Lamri (2018) reviewed various soft skills frameworks. In 2016, OECD released an overview of the key findings from the OECD Survey of Adult Skills (Kankaraš et al. 2016), which highlights the importance of soft skills in the labour market and discusses policy implications for developing these skills. 

Overall, despite the difficulty to agree on frameworks and terminologies, the relevance of soft skills for individual success in the workplace has been widely discussed in the literature. Numerous authors have called attention to the interplay between soft skills and other personal qualities to facilitate individual performance in the workplace or in general (Rychen and Salganik 2003; Kantrowitz 2005; Cimatti 2016; Ibrahim et al. 2017). Further, soft skills can be instrumental in improving work satisfaction and are associated with higher levels of engagement, productivity, and creativity in the workplace (Palumbo 2013; Feraco et al. 2023; the role of particular individual qualities or activities has been shown in numerous studies (Reysen et al. 2019; Feraco et al. 2023).

In terms of educability, Durlak et al. (2011) published a meta-analysis related to categories of self-emotional learning (SEL). This meta-analysis examined the effectiveness of school-based social and SEL programs in enhancing students’ skills, attitudes, prosocial behaviour, and academic performance. The researchers analysed data from 213 studies involving more than 270,000 students from kindergarten to high school. The results showed that students who participated in SEL programs had significantly better social and emotional skills, attitudes, and behaviour compared to their peers who did not participate in these programs. Additionally, the study found that students involved in SEL programs also had an 11 percentile-point gain in academic achievement. Another study considers soft skills through the prism of social, emotional, and behavioural skills (Soto et al. 2022). 

### 2.4. Differences and Commonalities between Hard and Soft Skills

It is important to have both hard and soft skills in order to be successful in the workplace. Research has shown that both types of skills are necessary and having a combination of the two leads to greater success (Rainsbury et al. 2002; Vasanthakumari 2019; Lyu and Liu 2021). For example, software development requires typically a variety of technical know-how and problem-solving capabilities (Groeneveld et al. 2021). For an individual to successfully complete such a task, he or she must often combine soft skills such as creativity and knowledge of various programming methods to come up with a successful solution. Designers must master computer software and physical tools to create prototypes as well as people skills to interact with clients or team members in collective design projects. 

Hard skills are necessary for specific knowledge-based tasks and are often taught in universities and technical schools. On the other hand, soft skills are often a better predictor of workplace success than hard skills, as they are essential for personal and interpersonal functioning (Hargood and Peckham 2017). Soft skills can help to identify candidates who have the necessary qualities to lead, manage, and collaborate, which are essential for a successful and productive workplace (Rainsbury et al. 2002). Additionally, soft skills are also important for customer service, which is a required and necessary component of most work environments.

Whereas the different terminologies highlight the various aspects of hard and soft skills, it is important to recognise that these skills often intersect and support one another in various contexts. As the literature continues to evolve, researchers are increasingly examining the interrelationships between hard and soft skills and their combined contribution to individual and organisational success. On many occasions, the differences between soft skills and hard skills are often difficult to discern. 

It is possible for an individual to have both strong soft and hard skills, and studies tend to show that it is the combination of both that increases an individual’s chances for success in the workforce by providing a well-rounded and competitive toolkit for employers (Rainsbury et al. 2002; Succi and Canovi 2020). Having a mixture of both types of skills is seen as a requirement for many positions.

When seeking to hire candidates, employers should consider the importance of both soft and hard skills. Although employers want typically to find someone who has technical expertise and qualifications, they should consider attributes such as creativity, communication, interpersonal skills, and problem-solving, as well (Lyu and Liu 2021). Research has shown that hard skills become obsolete more quickly than soft skills (Dominici 2019; Schultheiss and Backes-Gellner 2022), so employers should take into account the importance of both types of abilities when hiring. Furthermore, employers should also provide the necessary training and mentorship to ensure that their employees have the correct skillsets for the job (Succi and Canovi 2020).

Generally speaking, the criteria for determining whether a skill is soft or hard depend on the context in which the skill is used. Some researchers argued that soft skills are often seen as being more “Person-Centred” whereas hard skills are classified as “Task-Centred”, emphasising the need for individuals to be able to both interact with and help others (Rodríguez-Jiménez et al. 2021). As a result, soft skills are typically viewed as more important when it comes to interpersonal aspects of professional life such as communication, problem-solving, customer service, and teamwork, among others. Hard skills are generally evaluated and valued based on their effectiveness with regard to the completion of a specific task. 

Although hard and soft skills have different definitions and uses, they also overlap to some degree (Green 2011; Cinque 2016). For example, communication, although traditionally categorised as a soft skill, also involves technical aspects like data analysis and writing, using software to produce presentations. Similarly, interpersonal skills include specific knowledge about group behaviour and social codes, which could be seen as a hard skill (Bishop 2017). There exists an interdependent relationship between the two, with each trait enabling the other to succeed (Lyu and Liu 2021). As an example, hard skills such as accounting or designing require the support of certain soft skills, like communication and problem-solving, to truly display the potential of the hard skill. Additionally, numerous studies show a positive relationship between soft skills and hard skills performance (Kuzminov et al. 2019; Lyu and Liu 2021), suggesting the need for a synergistic combination of the two that can lead to successful job outcomes.

## 3. From Skills Theories to the Generic Skills Component Approach

### 3.1. Foundations for the Generic Skill Components Approach

Is the distinction between hard/soft useful? Is there, metaphorically, a scale of “hardness” of skills, like Mohs’ scale for the hardness of minerals, ranging from talc (very soft) to diamonds (very hard)? Numerous authors have raised the idea of a continuum from hard to soft skills passing by a vast mid-scale with semi-hard and semi-soft skills (see Andrews and Higson 2008; Clarke and Winch 2006; Dell’Aquila et al. 2017; Hendarman and Cantner 2018; Lyu and Liu 2021; Spencer and Spencer 1993; Rychen and Salganik 2003). Le Boterf (2000) suggests that skills are better understood as a continuum, with some skills containing both hard and soft components.

The generic skill components approach builds upon these recent findings, suggesting that all skills can be understood through a shared framework of five distinct components: knowledge, active cognition, conation, affection, and sensory-motor abilities. This integrated approach has the potential to reconcile the traditional distinction between hard and soft skills, providing a more comprehensive understanding of the complex nature of skills and their development.

### 3.2. Discrediting Skills as Discrete Entities

Working on a generic structure for all skills implies that skills are not discrete entities as such. We believe there is a necessity to clarify that aspect, before moving towards the construction of a generic skills approach. Consider the following arguments:

1. Overlapping and interrelated nature of skills: Skills are often interconnected and interdependent, making it difficult to clearly separate them into distinct categories. For example, the successful application of technical skills often depends on the presence of effective interpersonal skills, and vice versa (Kavé and Yafé 2014; Gardiner 2017). This overlap and interrelatedness challenges the idea that skills exist as discrete entities (Greenwood et al. 2013; Bean et al. 2018). 

2. Contextual factors: The relevance and importance of specific skills can vary depending on the context in which they are applied. This contextual variability can lead to differing interpretations and classifications of skills, further challenging the idea of skills as discrete and stable entities (Perkins and Salomon 1989; Hall and Magill 1995; Widdowson 1998).

3. Evolving skill requirements: The rapidly changing nature of work and technological advancements requires individuals to adapt continuously and develop new skills. As a result, the boundaries between different skill categories may become increasingly blurred as individuals are expected to possess a diverse and dynamic skillset (Dede 2010; Hargood and Peckham 2017; Dominici 2019).

4. Limitations of terminologies: The use of specific terminologies for hard and soft skills can sometimes oversimplify or constrain our understanding of the multidimensional nature of skills. By focusing on specific aspects or dimensions of skills, these terminologies may inadvertently perpetuate the idea that skills are discrete entities, rather than acknowledging the complex, interconnected permeable nature of skill development and application (Matteson et al. 2016; Lyu and Liu 2021).

The overlapping and interrelated nature of skills, the continuum perspective, contextual factors, evolving skill requirements, and the limitations of terminologies contribute to the difficulty of treating skills as discrete entities. Recognising these challenges can help researchers and practitioners develop more nuanced and integrative approaches to skill development and assessment. Building on this analysis, we believe there is a need for a unified approach to the structure of skills.

### 3.3. Using Goldstein and Hilgard’s Work as a Core Basis

The ambition to find a generic structure for skills is not new. Goldstein (1989) proposed a framework, with four components structuring any skill: cognitive, affective, motivational, and behavioural. In Goldstein’s, cognitive components involve the understanding and knowledge associated with a skill, such as problem-solving and analytical skills. Affective components involve emotions and attitudes, such as self-awareness and empathy. Motivational components involve the drive and determination to succeed, such as perseverance and ambition. Last, behavioural components involve the actual physical performance of a skill, such as hand-eye coordination and agility.

Although the literature is filled with definitions and discussions about skills, we choose in this article to use the work of Goldstein (1989) as a primary basis. His work, both theoretical and empirical, provides a comprehensive framework for understanding, designing, implementing, and evaluating skills development in organisations. 

Applying these four components to hard and soft skills, we can see that all skills are composed of the same elements, but with different weights depending on the context in which they are used. For example, a hard skill such as programming would require a higher level of cognitive ability but lower levels of affection. In contrast, a soft skill such as active listening would require a higher level of affection but lower levels of cognition. In that way, Goldstein’s framework seems a relevant basis to reconcile soft skills and hard skills. However, it is necessary to take a step back and take a closer look at Goldstein’s components.

Goldstein’s work relates to Hilgard’s (1980a) ‘Trilogy of Mind’, which describes human consciousness in terms of three main dimensions: cognition, conation, and affection. Hilgard (1975, 1980b, 1986) examines learning, personality, and hypnosis, and how they interact with one another to shape our understanding of the mind. Hilgard’s trilogy is itself based on the ‘Trilogy of Mind’ that Emmanuel Kant espoused.

Hilgard’s conception of these concepts differs from Goldstein’s:

- Cognition is the ability to think and solve problems, acquire information, and understand the world around us. It entails the processing of ideas and facts which allows the user to make better-informed decisions.
- Conation is the preferred pattern of actions and choices, integrating the results of cognitive processes to take action in order to achieve our objectives. It relies on the capacity to plan, as well as to monitor and evaluate our goal-driven performance.
- Affection is the ability to build and maintain relationships with others, stimulating social interaction and facilitating collaborative work. It involves the capacity to understand and empathise with others’ needs, as well as the ability to develop positive social networks.

In this approach, conation has a clear link with cognition and action, and we believe that, with some adaptations, it can be a promising way to apprehend motivational aspects, known as “volition” in some frameworks. Cognition should be treated as an active dynamic process. In this process, knowledge is acquired, used, transformed, and produced. It is however useful to distinguish the knowledge itself and the information-processing actions in which this knowledge is used. 

Affection as seen by Hilgard seems richer than what is envisioned by Goldstein and relates better to the concept of emotional intelligence (Goleman 1995). Goldstein underlines the importance of the body actually taking action. However, calling it behaviour might be confusing, regarding the extensive literature about behaviour, and the way behavioural psychology apprehends it. Following Goldstein’s definition, we believe sensory-motor abilities to be more appropriate as a component name.

Considering these adjustments, we propose the following revised framework for any skill, composed of five distinct components:

1. Knowledge includes both external knowledge or facts, such as technical job-related knowledge, as well as internal knowledge, such as memory (Bloch 2016; Zagzebski 2017).
2. Active cognition involves perceiving and processing information to form decisions and opinions, such as perception, attention, and judgement (Bickhard 1997). The analysis of the environment and the context falls under active cognition.
3. Conation is the component that describes preferences, motivations, and volitional components of behaviour. It is the drive or impulse to act and is often referred to as the “will” or “willingness” to act (Csikszentmihalyi 1990). We believe it goes beyond motivation as referred to by Goldstein.
4. Affection: Affection is the ability to empathise with and manage feelings in order to build and maintain relationships with others.
5. Sensory motor abilities: Sensory motor abilities refer to the ability to control and coordinate movements. This includes the ability to perceive, interpret, and respond to sensory input, as well as the ability to plan and execute movements. Examples of sensory-motor abilities include balance, coordination, and fine motor skills.

Using this framework, it becomes possible to describe both soft skills and hard skills in the same way. With time, we believe the distinction between both types of skills may become either obsolete or insufficient. Only the specific content and weight of each component would matter in order to describe a skill, to determine the overlap between two skills, or the transferability from one skill to another. 

### 3.4. Developing the Generic Skill Components Approach

The generic skill components approach aims to provide a comprehensive understanding of the structure and composition of any skill. This approach posits that all skills, whether hard or soft, can be understood in terms of five distinct components: knowledge, active cognition, conation, affection, and sensory-motor abilities. By examining these components and their interactions, we can gain a more in-depth understanding of the nature of skills and their development.

This approach is supported by previous research that has identified common elements across various types of skills. For example, Rychen and Salganik (2003) propose a model of key competencies that includes cognitive, intrapersonal, and interpersonal dimensions, which align with the active cognition, conation, and affection components of the generic skill components approach. Similarly, other studies highlight the importance of cognitive, affective, and behavioural processes in the development and application of both hard and soft skills (Parlamis and Monnot 2019; Soto et al. 2022). Our approach extends beyond existing models by incorporating sensory-motor abilities, which are often overlooked in discussions of skill development. This inclusion acknowledges the importance of physical and perceptual abilities in the successful application of many skills, particularly in fields such as sports, manufacturing, and healthcare.

This approach has several potential applications and implications for various fields, including education, training, and management. By understanding the generic components of skills, educators and trainers can develop more effective and holistic approaches to skill development, integrating both technical and non-technical components. In the workplace, a greater understanding of the generic composition of skills can help inform hiring decisions, performance evaluations, and employee development programs. If a skill has a major active cognition component, the resulting pedagogic engineering will be very different compared to a skill with a major knowledge component.

Further research is needed to refine and expand upon the generic skill components approach. Future studies could explore the interactions between the different components, as well as the impact of contextual factors on skill development and use. Indeed, the generic skill components approach highlights the importance of context in the development and application of skills, suggesting that educators and trainers should consider the specific environments in which their students or employees will be applying their skills. This may require the development of more context-specific training programs that focus on the unique challenges and opportunities presented by different work environments. Additionally, researchers could investigate the potential for more distinct skill categories and their implications for various domains.

### 3.5. Tentative Representation of the Generic Skills’ Components Framework

**Figure 1:**
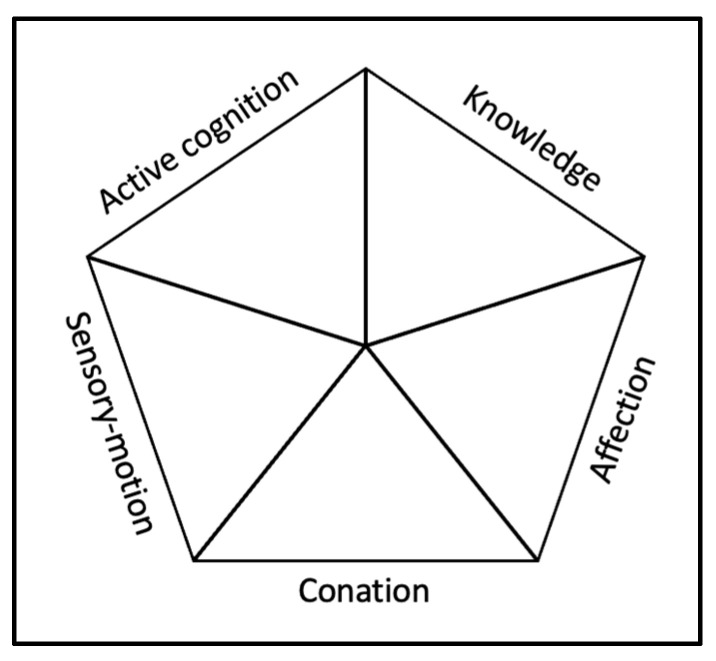
Visual representation of the generic skills’ components framework.

Although the approach needs to be further developed and tested empirically, we propose in this article an attempt at visual representation, displaying the five generic components in a diagram (see Figure 1). This diagram may be seen as a template to be used for skills description, as proposed later.

Our understanding of generic skills components would be that all components exist independently and need to be associated to create the necessary skill. This implies that they are not relative to each other, meaning that for a given skill, it is possible that all components are required at a very high level of mastery or development. Furthermore, conversely, for another skill, it is possible that all components are required at a very low level. In this manner, all types of combinations are possible, the point being that the necessity of one component at a high level does not determine the level of other components.

### 3.6. Tentative Representation of Skills Composition Using the Framework

Below, we propose three examples of using the framework to represent skills: oral communication, Python programming, and logical analysis. At this stage, the assessment is very basic, as it results in a consensus among the authors, having both theoretical and empirical experience in skills expertise. These specific cases of skill descriptions will need to be challenged in order to be considered consensual, but the purpose of this section is rather to show the possibilities offered by the generic skills’ components approach. For each skill, we propose:

- A visual representation based on the generic skills’ components framework (see Figure 1);
- A rating from 1 (low) to 5 (high) for each component;
- An explanation of the importance given to each component in the context of the skill;
- A suggestion of a training program detailed for each component.

(A) Example 1: Oral communication

**Figure 2:**
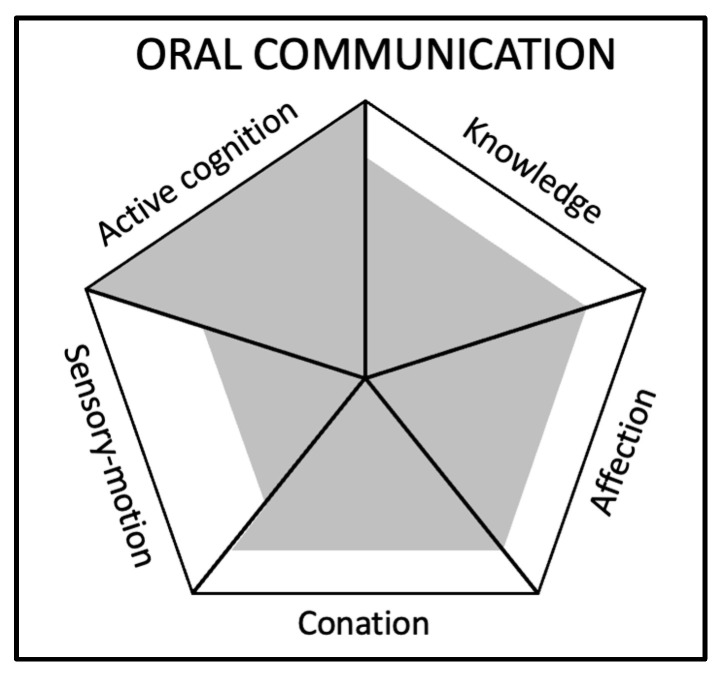
Visual representation of the generic skills components’ framework for the skill ‘Oral communication’.

For the skill “oral communication”, which is usually referred to as a soft skill, we describe below on a scale of importance of 1 to 5 for each component, the composition for each component (see Figure 2):

1. Knowledge: 4/5—Knowledge is essential for effective oral communication, as it involves understanding the topic being discussed, the context, and the audience. Having a solid grasp of the subject matter, as well as cultural and social norms, allows the speaker to convey messages accurately and effectively. Additionally, internal knowledge helps the speaker to convey relevant information and experiences to support their points.
2. Active cognition: 5/5—Active cognition is crucial for oral communication, as it involves perceiving and processing information in real-time. Effective oral communication requires the speaker to pay attention to the audience, adapt the message based on audience reactions, and make judgments about what information to share and how to present it. It also involves critical thinking and problem-solving skills, as the speaker may need to respond to questions or objections from the audience.
3. Conation: 4/5—Trait extraversion can support oral communication because it motivates the speaker to engage with the audience and present the message confidently and persuasively. A strong willingness to act can also help the speaker overcome any anxiety related to speaking in front of others.
4. Affection: 4/5—The ability to empathise with and manage emotions is important for connecting with the audience and creating a positive atmosphere during oral communication. Understanding the emotional state of the audience can help the speaker adjust their/his/her tone and approach while managing their/his/her own emotions can ensure a calm and composed delivery. Additionally, being able to express warmth and enthusiasm can make the message more engaging and persuasive.
5. Sensory motor abilities: 3/5—Although not as critical as other components, sensory-motor abilities still play a role in oral communication. The ability to control and coordinate movements, such as gestures and facial expressions, can help the speaker convey a message more effectively and make a stronger impression on the audience. Proper posture, eye contact, and voice modulation are also important aspects of oral communication that rely on sensory-motor abilities.

It is interesting to observe that using the framework, it appears that all components are relevant to the skill of oral communication. This example shows the value of such skills that can be underestimated in their complexity.

To develop the skill of oral communication using this framework, a pedagogical program could be designed as follows:

1. Knowledge:
  - Provide learners with the necessary knowledge related to the subject matter they will be communicating, whether it is through lectures, research, or reading.
  - Encourage learners to integrate this knowledge into their communication to increase their credibility and effectiveness.
2. Active cognition:
  - Provide learners with opportunities to practise active listening and critical thinking to understand better the needs of their audience and adapt their communication accordingly.
  - Encourage learners to use visual aids or other communication tools to increase their impact and effectiveness.
3. Conation:
  - Provide learners with opportunities to practise oral communication in a safe and supportive environment, such as through role-playing or group discussions.
  - Encourage learners to take risks and learn from their mistakes, building their confidence and willingness to communicate effectively.
4. Affection:
  - Integrate exercises and activities that promote empathy and emotional intelligence, such as reflecting on the emotional impact of communication or practising active listening.
  - Encourage learners to build positive relationships with their audience, as this can enhance their effectiveness as communicators.
5. Sensory motor abilities:
  - Provide learners with opportunities to practise their oral communication skills, such as pronunciation, articulation and voice projection exercises.
  - Encourage learners to practise clear and effective body language to enhance their overall communication skills.

Overall, a training program created according to the skills generic components approach should emphasise the importance of all five components of the framework and provide learners with the opportunity to develop each one in a holistic and integrated manner. By focusing on all the aspects of oral communication, learners can develop the skills they need to communicate effectively and build positive, meaningful relationships with those around them.

(B) Example 2: Python programming

**Figure 3:**
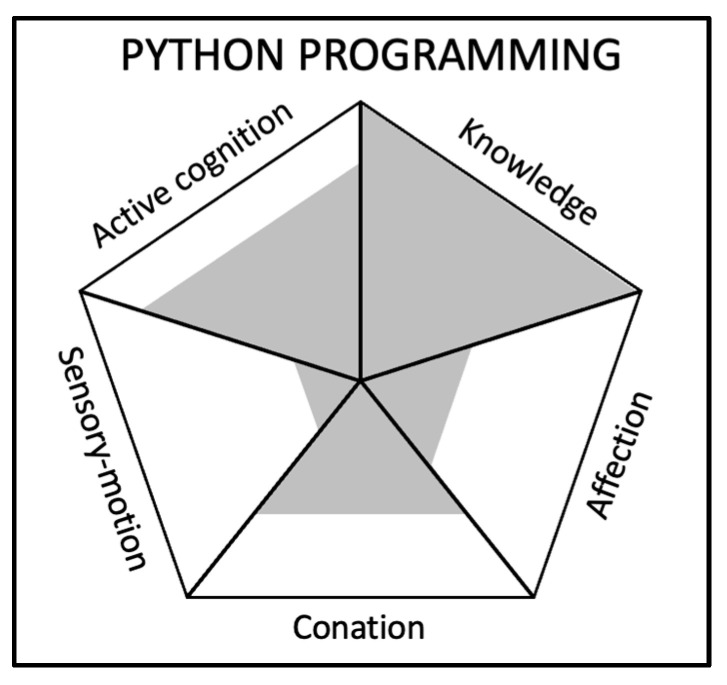
Visual representation of the generic skills’ components framework for the skill “Python programming”.

For the skill “Python programming”, which is usually referred to as a hard skill, we indicate the importance of each component on a 5-point scale, and describe, the composition for each component (see Figure 3):

1. Knowledge: 5/5—Knowledge is crucial for Python programming, as it involves understanding the syntax, functions, libraries, and best practices in the language. A programmer must be knowledgeable about programming concepts, algorithms, and data structures to effectively use Python in various applications. This includes both external knowledge, such as learning from resources and documentation, and internal knowledge, such as remembering previously learned concepts and experiences.
2. Active Cognition: 4/5—Active cognition plays an important role in Python programming, as it involves perceiving and processing information to form decisions and opinions. This includes understanding the problem being solved, designing an appropriate solution, and troubleshooting any issues that arise during coding. Active cognition also involves adapting to new programming paradigms, tools, and techniques.
3. Conation: 3/5—Conation is moderately important in Python programming. Although having the motivation and willingness to learn and improve one’s programming skills is important, it may not be the primary driver for success in this field. However, showing perseverance, and having a strong drive to problem-solve, debug, and optimise code can contribute to better overall performance and growth as a programmer.
4. Affection: 2/5—Affection has a lower importance in Python programming compared to other components. While empathy and emotional intelligence may not directly contribute to programming skills, they can still play a role in building positive relationships with teammates or clients, understanding user needs, and contributing to a healthy work environment. Good communication and collaboration skills can also help when working on projects with others.
5. Sensory Motor Abilities: 1/5—Sensory motor abilities have minimal importance in Python programming. While basic motor skills are needed for typing and using a computer, the primary focus in programming is on cognitive and knowledge-based skills. However, maintaining proper ergonomics and posture while working at a computer can help prevent physical strain and promote overall well-being.

It is interesting to observe that using the framework, it appears that active cognition and knowledge seem to be the most important components for the skill of Python programming. However, conation is not to be underestimated. Knowledge is commonly associated with hard skills, whereas active cognition and conation are commonly associated with soft skills. Although knowledge seems more important than the other components, we believe the importance of other components is generally underestimated when considering Python programming as a hard skill, as context matters. This example shows value for such skills that are unfairly considered hard skills with little to no consideration for the potential complexity of the context, or the motivation of the programmer.

To develop the skill of Python programming using the framework of the five components, a pedagogical approach can be designed as follows:

1. Knowledge:
  - Begin with teaching the fundamentals of Python, such as data types, variables, control structures, and functions, through a combination of lectures, reading materials, and online resources.
  - Introduce more advanced concepts, such as object-oriented programming, error handling, and file I/O, as students progress.
  - Teach students about commonly used Python libraries and their applications in various domains.
  - Assign small projects or exercises at the end of each topic to reinforce learning.
2. Active Cognition:
  - Encourage students to practise problem-solving using Python by assigning coding challenges and puzzles that require critical thinking and decision-making.
  - Provide opportunities for peer programming, where students collaborate and exchange ideas to solve problems.
  - Organise regular code review sessions to help students learn from each other’s solutions and improve their problem-solving strategies.
3. Conation:
  - Set clear expectations and learning goals for students to motivate them to learn and practice Python programming.
  - Offer regular feedback and support throughout the learning process to help students stay engaged and committed.
  - Encourage students to participate in coding competitions, hackathons, or open-source projects to build their confidence in Python programming.
4. Affection:
  - Foster a supportive learning environment in which students can openly discuss their challenges and successes in Python programming.
  - Encourage students to work in teams for some projects, which will help them develop shared (and hopefully positive) emotional experiences.
  - Provide opportunities for mentorship or tutoring, where more experienced students can assist their peers in learning Python programming.
5. Sensory Motor Abilities:
  Although sensory-motor abilities are not directly relevant to Python programming, promoting healthy computer use habits can indirectly support skill use.
  - Teach students about ergonomics and the importance of regular breaks to prevent strain and fatigue while working on a computer.
  - Encourage students to engage in physical activities or exercises to maintain overall well-being, which can have a positive impact on their cognitive abilities.

By incorporating these strategies in a Python programming course or training program, learners can develop the required skills while addressing all components of the pedagogical framework.

(C) Example 3: Logical analysis

**Figure 4:**
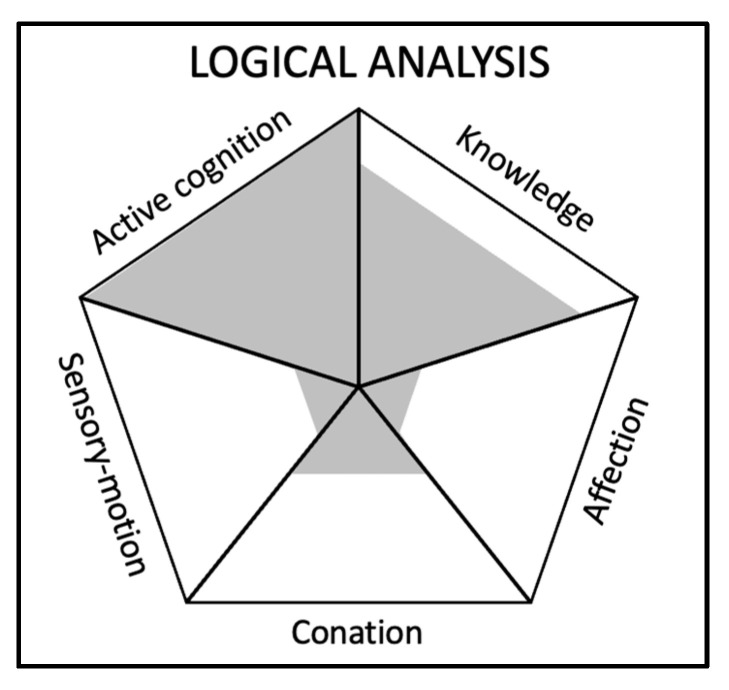
Visual representation of the generic skills’ components framework for the skill “Logical analysis”.

For the skill “logical analysis”, which is ambiguously considered as a soft skill or a hard skill depending on the situation, we describe below on a scale of importance of 1 to 5 for each component, the composition for each component (see Figure 4):

1. Knowledge: 4/5—Logical analysis requires a solid foundation of knowledge about the subject matter being analysed. This includes understanding key concepts, principles, and relationships within the domain. For example, analysing a scientific argument requires knowledge of the relevant scientific facts and theories. However, the ability to apply logic and reasoning is also essential, so knowledge alone is not enough for logical analysis.
2. Active cognition: 5/5—Active cognition is crucial in logical analysis, as it involves the ability to perceive and process information, identify patterns and relationships, and evaluate the validity of arguments. This includes skills such as critical thinking, problem-solving, and decision-making. Active cognition allows individuals to analyse situations, evaluate evidence, and form sound judgments based on logical reasoning.
3. Conation: 2/5—Whereas motivation and the willingness to engage in logical analysis are necessary, conation also plays a supporting role through perseverance and perfectionism, which ensures that individuals are committed to the process of logical analysis and persist in their efforts to reach accurate conclusions.
4. Affection: 1/5—Affection, as defined by empathy and emotional management, is not a central component of logical analysis. Logical analysis focuses primarily on rational thinking and objective evaluation of evidence, rather than emotional connections and relationships. However, having a certain level of emotional intelligence can help individuals avoid potential biases and maintain objectivity in the analysis.
5. Sensory motor abilities: 1/5—Sensory motor abilities are not directly relevant to the skill of logical analysis, as logical analysis is a cognitive process that does not rely on physical movement or sensory input. Although sensory-motor abilities may be necessary for other skills, they do not play a significant role in logical analysis.

It is interesting to observe that using the framework, it appears that active cognition and knowledge seem to be the most important components for the skill of logical analysis. Knowledge is commonly associated with hard skills, whereas active cognition is commonly associated with soft skills. The dominance of these two components could explain why it seems complicated to categorise logical analysis as a soft or hard skill. This example shows the value of such skills that cannot be consensually categorised.

To develop the skill of logical analysis using the framework based on the five components, a pedagogical approach can be designed as follows:

1. Knowledge:
  - Begin by teaching the basic logical concepts, such as premises, conclusions, and logical fallacies.
  - Teach various types of logical arguments and structures (e.g., deductive, inductive, and abductive reasoning).
  - Provide examples and case studies to illustrate different logical principles and argumentation styles.
2. Active Cognition:
  - Engage students in debates or discussions to practise identifying and evaluating arguments.
  - Provide exercises that require students to identify logical fallacies or errors in reasoning.
  - Engage reflection and self-assessment to help students recognise their own biases and assumptions.
3. Conation:
  - Set clear goals and expectations for students’ progress in developing logical analysis skills.
  - Provide regular feedback and encouragement to help students stay committed and motivated.
  - Create opportunities for students to collaborate and share their learning experiences with peers.
4. Affection:
  - Teach students how to present their logical analyses effectively and persuasively, while considering the perspectives and emotions of their audience.
  - Encourage empathy and active listening during debates and discussions to foster a more open and collaborative learning environment.
5. Sensory Motor Abilities:
  - Present information and materials in a clear, visually appealing manner to facilitate understanding.
  - Encourage students to take notes or create visual representations (such as diagrams or flowcharts) to help organise and process information.

By addressing each component of the generic framework, this pedagogical approach provides a comprehensive and structured method for developing logical analysis skills in students.

## 4. Limitations and Opportunities

Skills have traditionally been defined as a set of competencies or abilities that an individual has, such as problem-solving, analytical thinking, and communication. However, this definition is problematic because it treats skills as discrete entities; this fails to account for the influence of contextual factors on how skills are used in practice. For example, a skill such as communication may be used differently in diverse contexts, with different levels of success. Further, there may be no such thing as a completely “generic” skill—one that functions equally well in all contexts. In short, the idea of skills as abstract entities is a misleading oversimplification.

The definition of skills as abstract entities has a wide range of implications. It ignores the role of context in how skills are applied, which in turn can lead to an over-emphasis on the individual’s capabilities and an under-emphasis on environmental conditions (Widdowson 1998). This can lead to a focus on individual differences instead of a collective approach; this in turn can lead to a narrow focus on the individual and an inability to identify external influences on skill use. Further, it can lead to a teleological approach (González Galli et al. 2020), whereby skills are thought to be automatically “transmitted” to the context in which they will be used, without regard to the idiosyncrasies of that context. Finally, it can lead to a focus on skills as an end in themselves, instead of collectively as part of a much larger system.

A systems-based perspective goes beyond the traditional concept of skills as abstract entities and instead focuses on the way in which skills develop within specific contexts, thus treating them not as static entities, but as part of an interactive, evolving system. Through this perspective, the influence of context on skill use is fully acknowledged, with multiple factors—such as culture, power dynamics, and social norms—being taken into account. Therefore, this approach enables the concept of skills to be seen as part of a larger system of behaviour and learning, which is essential to understanding how skills can be effectively developed, practised, and utilised.

Indeed, the scientific literature has challenged the definition of skills as abstract entities and instead advocated for a systems-based approach that acknowledges the role of context in how skills are applied (Tracey et al. 1995; Le Boterf 2000; Sih et al. 2019). However, if skills did not exist, then only knowledge would matter a priori. 

Knowledge alone does not lead to successful interactions with others; skill plays an integral role in the development of successful social behaviours (Boyle et al. 2017; Rios et al. 2020). Further, this research indicates that even if a person has a great deal of knowledge, it is not enough to produce the desired results unless they can put the knowledge into practice. Skills need to exist in order to allow professionals, educators, and clinicians to work on isolated and specific constructs, even if variable and not perfect as such. In our contribution, we see the generic components approach as a way to redefine the concept of skill, by embedding environmental factors in cognitive, conative, and affective dimensions.

Although our generic skill framework provides the basis for further developments, it is important to note that other approaches may need to be considered to provide a more comprehensive understanding of the concept of skill in various contexts.

## 5. Conclusions

This article has explored the definitions, categories, and impact of both hard and soft skills in order to gain an understanding of the generic composition of any skill. It found that both must be viewed as complementary elements comprising a successful performance and that hard skills are objective and quantifiable capabilities that are easily measured, whereas soft skills are non-technical, interpersonal, and visual qualities that are often learned through experience. Although the two types of skills are often classified separately, understanding their interdependence can help create a more comprehensive skill set. Strategic thinking and action, skills that cut across both soft and hard skills, are essential for making effective decisions.

Research on skills reveals that hard and soft skills often overlap, with various components being shared between them. As such, there is a need to recognise the different components of any skill to develop individuals efficiently and effectively. The generic components proposed in this article open the way to discuss the common ground between hard skills and soft skills, and more broadly the generic composition of any skill. More research is needed to refine the approach on this topic, but it seems a greater understanding of the generic composition of skills can help inform professional, educational, and clinical practices.
